# On the Sanitary and Social Condition of the English Poor

**Published:** 1855-06

**Authors:** 


### ON THE SANITARY AND SOCIAL CONDITION OF THE ENGLISH POOR.

By a PRACTISING PHYSICIAN.

No. II.

To return from the digression into which I was almost unconsciously drawn towards the close of my last communication, I would remark that, while the inhabitants of increasing cities and towns have gone on outstripping by far the inhabitants of rural districts in all matters pertaining to education and political influence, they have in the construction of their dwellings departed from many of those simple and instinctive rules for the preservation of health, which persons of much ruder and of more uncultivated intellects have made it a habit to follow. This fact admits of several illustrations.

I have often heard sensitive and kind-hearted people descant largely on the hardships endured by wandering tribes and vagrants, such as the gipsy tribes and professed beggars, who sleep in tents or sheds, and have no regular home; but I can safely say, both as a physiologist and an observer, that the sanitary condition of the gipsy at all events is infinitely superior to that of three-fourths of our labouring population in populous towns. Indeed, after having made extensive inquiries as to the general health of the English gipsy, I can say that there is no class of the community that represents better the *mens sana in corpore sano*. Bodily deformities are rare amongst the gipsies; mental disease is almost unknown, with the exception of the second childishness of the seventh stage of life.

They escape remarkably from epidemics and visitations. Their women give easy birth to healthy children; and their men and their women attain, as a general rule, to a ripe old age. In them what has been properly called "premature old age," is really unknown. And if some form of accident leading to a fatal end, as a contagious distemper or a physical injury does not step in the way to carry them off, they live on very commonly far beyond the term of three-score years and ten, retaining their physical and mental faculties with remarkable vigour and to the last. It will be said by some, that this marked good health in the gipsy race arises from what is called their hardiness of constitution. But this is only verbiage; for what is hardiness of constitution but mere good health?—and what is there in a gipsy *per se* more than in a city tailor or a Birmingham knife-polisher, that the one should live in good health, and the other should be the constant and inevitable victim of disease? Has not a gipsy "hands, organs, dimensions, senses, affections, passions? fed with the same food, hurt with the same weapons, warmed and cooled by the same winter and summer" as another man is? if you "prick him, does he not bleed? if you tickle him, does he not laugh? if you poison him, does he not die?" The simple truth is, that to cloak our own absurdities, we invent absurd apologies. The whole secret of the gipsy health, and of the robustness of many other nomade tribes, arises from an instinctive adherence to those natural laws, the philosophy of which the physiologist is now trying, with great labour and difficulty, to discover and to teach.

Let me illustrate this point in a few sentences.

In proportion as individuals are crowded together in small rooms, so in proportion do they find it difficult to support a healthy degree of animal heat. The reason of this is, that the temperature of the animal body is not really supported by the warmth of the air around it, but by the process of respiration or the breathing of pure air. In plain terms, the body contains its own furnace and makes its own heat; takes in its own fuel for combustion by the stomach; blows its own fire by the lungs, and casts off its own cinders and smoke by its excreting organs. The only advantage of a warm air, therefore, is, that it prevents a rapid loss of heat from the body; and hence woollen clothes, which are bad conductors of heat, keep the body as warm as a warm atmosphere would do; but the act of surrounding the body by a warm medium is of secondary importance; and a man would grow cold in a hot bath if he were not at the same time supplied with pure air for respiration, and with food for the manufacture of new blood. The simple ignorance of this simple fact leads to innumerable errors in daily practice. Small rooms are notoriously cold rooms, and when they are closed up fires in them burn with difficulty; and persons living in them become chilly, weak, and miserable. To make matters still worse, the occupants of such apartments do all they can to shut out common air. They barricade the windows; they hang up counterpanes or tablecloths before the doors; they half stifle themselves, in fact; and, without succeeding at all in obtaining a genial and refreshing warmth, they succeed admirably in inducing a dry feverish excitement, which departs instantly on the slightest cause. In simple words, by this act of ignorance they depress and partially extinguish the vital fire, upon which their very existence depends.

Compare these proceedings with the habits and customs of the gipsy in his tent. In pitching his temporary home, this nomade seeks only for three conditions: a dry piece of ground; shelter from the mechanical effects of the winds; and a waterproof covering. With these advantages at hand, he requires but sufficient straw for his bed, and woollen stuff to wrap himself in, and he becomes the most perfect specimen extant of a warm-blooded animal, provided, of course, that he has a sufficiency of nutritious food to support the combustion of his body.

Persons who are accustomed to live in the open air are always peculiarly susceptible to the depressing influence of a confined atmosphere. The practitioner of medicine residing in an agricultural district, soon learns this lesson, and he often cannot succeed in keeping his patients in bed, even when their state requires absolute rest. Your true English farmer is very unmanageable in this respect; but he is an absolute slave in this regard in comparison with your true English gipsy. Two years ago I attended a gipsy-boy on a large common near London. He had met with an accident, which had injured his spine, and his lower limbs were paralyzed. A medical friend who had seen the poor lad before me, very kindly and considerately sent up to one of the most famous London hospitals, and took great trouble in getting him admitted as an in-patient. This kind act proved useless,—the confinement of the wards was too oppressive for the child of nature. In the course of a week he suffered so much that he became hypochondriacal, and protested that he could not live another day in such a place. His complaints had really a physical cause, and it was considered most prudent to allow him to return to his bed of straw and canvas shelter. Here, for month after month, with numerous ulcers on every part of his body that touched his hard bed, he lingered, defying death in a manner which I have never witnessed. His position to some minds would seem almost horrible, but to have changed it would have been an ignorant piece of charity, and would have brought on his death speedily. He lived long, despite disease and suffering, because he imbibed in liberal proportions the prime agents on which alone life depends, nourishments *minus* luxuries, and pure air.

I notice often that people who live in confined rooms do themselves a great deal of harm by a childish and ignorant fear of what are called draughts. To keep out these evil spirits they block up every chink, and half suffocate themselves in consequence. There can be no doubt that if a limited current of air falls upon the body it will in some cases give rise to cold; but the philosophy of prevention does not consist in shutting out the air, but in letting it in freely at every point, that the body may be equally bathed in its life sustaining principle.

The evils of a confined atmosphere, suffered by the working poor, do not always arise from over-crowding. In some establishments, for example, where the men engaged are great sufferers from a deficient supply of pure air, the space allowed is ample, since the machines at which they work are too large to allow of the huddling-together process. In these instances, a shameful and sinful neglect of ventilation is the only, or at least the main, cause of the bodily depression. In a large printing establishment which I visited some months since, this fact was painfully evident. The printers employed were more like shadows than men; and if the chapel ghost, which Benjamin Franklin tells us of, had been called for in this building, any one of the occupants might have answered the summons without impropriety. Now the rooms in which these workmen were occupied were sufficiently large, and as the "cases" took up a great amount of space, there was no possibility of over-crowding. Whence then the impurity of these apartments? Whence the heated, sickening, disease-nourishing condition of the atmosphere? Solely in the absolute deficiency of any attempt at ventilation. I venture to say, that these rooms had never been thoroughly purified, and recharged with fresh air since the day on which they were built. They were stagnant pools of gas,—a mixture of human breath and of such air as was by accident admitted. The atmospheric pressure and the laws of gaseous diffusion were here counteracted in the most scientific manner, and if they were not stopped altogether, it was from no fault on the part of the owners of these gigantic grotto *del cane* premises.

In some manufactories the evils of bad ventilation are even more striking than in the above example. This obtains in manufactories where a copious amount of heat is evolved, attended perhaps with a considerable diffusion of watery vapour, and where ventilation is quite disregarded. It is curious to observe, that in some cases of this class, the ignorance displayed in regard to ventilation is not merely hurtful to the artisans, but is also an obstacle to the rapid accomplishment of the business that is carried on. Thus in buildings erected for the purpose of drying moist fabrics, at a very high temperature, the evaporation is often half checked by the want of sufficient openings in the walls or roofs of the drying-rooms. And the air of these rooms, despite its heat, is surcharged with watery vapour. I once endeavoured to explain to an overseer of one of these places, how mischievous a principle they were working upon, both in regard to the health of the men who had to turn the drying fabrics, and in regard to the success of the process that was being then pursued. A contemptuous laugh was the only response. The overseer was a *practical* man, and was not to be cajoled by common-sense theories.

